# Y90 Radioembolization in chemo-refractory metastastic, liver dominant colorectal cancer patients: outcome assessment applying a predictive scoring system

**DOI:** 10.1186/s12885-016-2549-x

**Published:** 2016-07-20

**Authors:** Robert Damm, Ricarda Seidensticker, Gerhard Ulrich, Leonie Breier, Ingo G. Steffen, Max Seidensticker, Benjamin Garlipp, Konrad Mohnike, Maciej Pech, Holger Amthauer, Jens Ricke

**Affiliations:** Department of Radiology and Nuclear Medicine, University of Magdeburg, Leipziger Str. 44, 39120 Magdeburg, Germany; Deutsche Akademie für Mikrotherapie e.V., Magdeburg, Germany; Department of General and Visceral Surgery, University of Magdeburg, Magdeburg, Germany

**Keywords:** Y90 radioembolization, Colorectal cancer, Liver metastases, Salvage patients, Prognostic score

## Abstract

**Background:**

In treatment-refractory liver dominant metastatic colorectal cancer, the role of liver directed therapies still is unclear. We sought to determine a prognostic score for Y90 radioembolization in these patients.

**Methods:**

We analyzed 106 patients with refractory liver dominant mCRC who had undergone a total of 178 Y90 radioembolizations with resin microspheres was collected. Potential factors influencing survival were analyzed using a Cox regression. The Log rank test served to establish prognostic factors and to form a clinical score for outcome prediction after Y90 radioembolization.

**Results:**

Median survival of all patients was 6.7 months. Neither age nor prior surgical or systemic therapy nor metastatic spread had an effect on survival. In contrast, hepatic tumor load, Karnofsky index as well as CEA and CA19-9 serums level had a significant influence (*p* < 0.001, *p* = 0.037, *p* = 0.023 and *p* < 0.001, respectively). These three factors formed a score with 1 point each for tumor load >20 %, CEA >130 ng/ml or CA19-9 > 200U/ml and Karnofsky index <80 %. Patients with a score of 0 and 1 displayed a median OS of 10.4 months. Patients with a score of 2 and 3 demonstrated a median OS of 5.1 months only (*p* < 0.001).

**Conclusion:**

Overaggressive patient selection for Y90 radioembolization of liver dominant chemorefractory mCRC is of questionable benefit. A scoring system comprising hepatic tumor load, CEA and CA19-9 serum levels and Karnofsky index (TuCK-score) may support an improved patient selection. In our cohort of liver only versus liver dominant disease, extrahepatic lung or lymphatic metastases did not significantly alter the prognosis.

## Background

In treatment-refractory liver dominant metastatic colorectal cancer, the role of liver directed therapies still is unclear. For Yttrium 90 (Y90) radioembolization (RE), objective response rates between 33 and 48 % have been published when applied in second line [1, 2], and between 10 and 48 % in third line [3–6]. In a refractory third line setting Seidensticker et al. reported improved survival in a match pair study of 3.5 vs 8.3 months^6^. Hendlisz et al. randomized between 5-fluorouracil (5-FU) +/− Y90 radioembolization in refractory third line patients and demonstrated a survival benefit of 10 versus 7.3 months, as well as a significant progression free survival (PFS) improvement of 4.3 vs 2.1 months. The latter study led to inclusion of Y90 radioembolization in the ESMO guidelines for colorectal cancer, however with a low strength of recommendation (IV, B) [7].

In clinical practice, treatment recommendations for refractory patients are challenging. Many patients present with advanced tumor load in a biologically unfavorable state of disease progression, potentially aggravated by comorbidities and a poor performance status. Palliation in such patients must balance the patient´s desire for life prolongation and an acceptable quality of life to withstand the hazards of aggressive treatments. Hence, a careful patient selection is of utmost importance. For Y90 radioembolization, no adequate predictive factors have been published in treatment refractory colorectal cancer patients yet, baring the risk of overtreatment as a result of inappropriate patient selection.

In the study described herein we analyzed a cohort of 106 patients with chemo-refractory, liver dominant colorectal cancer undergoing Y90 radioembolization at our institution. We sought to determine predictive factors to aid a responsible patient selection balancing the potential survival benefits against the inadvertent risk of an aggressive liver directed therapy.

## Methods

### Study design and eligibility criteria

Our patient database was reviewed for patients with colorectal cancer liver metastases undergoing Y90-radioembolization in our department between 2006 and 2010. We collected retrospective data on prior surgical or systemic treatments, disease spread, clinical performance, tumor markers and survival. All patients had been scheduled for routine follow-up every 3 months at our department including a documentation of their clinical performance, disease response in Computed tomography (CT)/magnetic resonance imaging (MRI) and laboratory values. We selected all patients who met the following criteria:

- liver metastases of colorectal carcinoma,
- admitted and eligible for Y90-radioembolization,
- salvage situation (either refractory to all accepted chemotherapy regimen at the time of admission or refusal of or non-eligibility to further systemic therapies after at least one cycle).

### Patient cohort

We included a total of 106 salvage patients with liver metastases from colorectal cancer (70 male, 36 female; mean age 61.9 years). All patients had failed at least one chemotherapy regimen; the median number of chemotherapy lines applied was 3. 26 % of the patients had failed four or more lines of systemic therapy. About half of the patients had received bevacizumab (*n* = 67) and/or cetuximab (*n* = 51) prior to radioembolization. 27 presented under maintenance therapy with capecitabine. Other cytotoxic regimen such as a combination of mitomycin and 5-FU were applied to 7 patients before admittance to Y90 radioembolization.

In 22 patients, a contraindication such as bone marrow depression or unwillingness to receive further chemotherapies (mostly as a result of previous toxicity such as polyneuropathy) led to discontinuation of systemic therapies.

Thirty patients had previously undergone hepatic resection or radiofrequency ablation before Y90 radioembolization. Whereas 86 patients presented liver only disease, 30 patients demonstrated extrahepatic tumor spread such as lymph node metastases (*n* = 17), lung metastases (*n* = 16, with 15 patients displaying more than one pulmonary metastases) and bone metastases (*n* = 4). Further details of patient characteristics are displayed in Table 1.

**Table 1:** Patient characteristics

Patient characteristics		
Female	*n* = 36	33,9 %
Male	*n* = 70	66,1 %
Age (median (range), years)	62.5 (33.0 -76.0)	
Karnofsky index (median (range), %)	80 (60–100)	
Pretreatment characteristics		
Prior resection or radiofrequency ablation	*n* = 30	28 %
Prior chemotherapy agents		
Oxaliplatin (+5-fluorouracil)	*n* = 79	75 %
Irinotecan (+5-fluorouracil)	*n* = 89	84 %
Capecitabine	*n* = 27	25 %
Bevacizumab	*n* = 67	63 %
Cetuximab	*n* = 51	48 %
Other	*n* = 7	7 %
Overall chemotherapy lines (median (range))	3 (1–5)	
1^st^ Line	*n* = 9	8 %
2^nd^ Line	*n* = 35	33 %
3^rd^ Line	*n* = 34	32 %
4^th^ Line and beyond	*n* = 28	26 %
Tumor characteristics		
UICC stage (median (range))	4 (1–4)	
Grading (median (range))	2 (1–4)	
Synchronous lymphatic metastases	*n* = 80	75 %
Extrahepatic tumor sites prior to radioembolization	*n* = 30	28 %
Solitary/oligonodular lung metastases	*n* = 1	1 %
Diffuse lung metastases	*n* = 15	14 %
Lymphatic metastases	*n* = 17	16 %
Bone metastases	*n* = 3	3 %
Hepatic tumor load (median (range), %)	15.7 (1.0–63.0)	
CEA serum level (median (range), ng/ml)	130.1 (2.7–8713.3)	
CA19-9 serum level (median (range), U/ml)	192.7 (0.3–32206.0)	
Radioembolization procedures	*n* = 178	100 %
Bilobar (total liver)	*n* = 12	7 %
Unilobar	*n* = 52	30 %
Sequential lobar	*n* = 114	64 %
Treatment sessions per patient (median (range))	2 (1–5)	
Total activity per patient (median (range), MBq)	1725.0 (200.0–3650)	

### Clinical evaluation and radioembolization technique

All patients underwent a thorough clinical examination prior to radioembolization including a physical examination, laboratory tests and cross-sectional imaging including MRI with hepatocyte-specific contrast agent Gd-EOB-DTPA (Primovist®, Bayer HealthCare, Leverkusen, Germany).

The technique of Y90 radioembolization has been described in detailed elsewhere [8].

All patient scheduled for radioembolization received an initial evaluation angiography. The work up included coil or plug embolization of visceral collaterals if appropriate. After the test infusion of Technecium-99 m macro-aggregated albumin (Tc-99 m MAA, LyoMAA, Covidien, Neustadt, Germany) to the liver arteries, a scintigraphy including a SPECT-CT was performed to rule out extrahepatic accumulation or inadvertent lung shunting. In the latter case, a lung shunt above 10 % led to a dose reduction as specified by the summary of product characteristics. Dosimetry was performed applying the body-surface area model [9].

Liver metastases were treated exclusively employing resin microspheres (SIR-Spheres®, Sirtex Medical, Lane Cove, Australia) labeled with beta-emitter Yttrium-90 (half-life 64 h; mean energy 0.96 MeV). The catheter position during Y90 application was identical to the test bolus of Tc-99 m MAA during the evaluation. Starting in 2007, radioembolization was typically partitioned in sequential therapies for each liver lobe at an interval of 4 to 6 weeks. In patients presenting with disease limited to one liver lobe, Y90 spheres were applied in the according lobe only with a dose calculation adopted to the reduced liver volume [10]. Before 2007, we exclusively performed whole liver treatments.

All patients gave written informed consent to both radioembolization as well as the scientific use of their personal data. The institutional ethics committee deemed a dedicated ethics vote unnecessary for the present analysis. This scientific paper has been written according to the reporting standards for radioembolization [11].

### Data collection

Follow-up data was acquired until May 2013. The patient database contained a prospective data set including therapies prior to presentation at our institution (surgical resection, chemotherapy and combined immunochemotherapy), tumor markers (Carcinoembryonic antigen, CEA and Cancer antigen 19–9, CA19-9 serum levels), individual patient characteristics (age, sex, Karnofsky index) and imaging aspects (initial staging, tumor distribution and hepatic tumor load). Finally, details of Y90 radioembolization (applied activity, number of treatment sessions and more) were included. Side effects were defined and categorized by the Common Terminology Criteria for Adverse Events (CTCAE, Version 4.03).

### Statistical analysis

SPSS 21.0 (IBM®, New York, USA) was used for the entire analysis. Descriptive analysis was computed with median and range of continuous variables as well as frequencies of nominal data. Univariate stepwise Cox regression analysis was used to determine factors influencing patient survival. Any factor with a tendency towards significance (*p* ≤ 0.1) was included in a multivariate Cox proportional hazard model. Variables demonstrating a significant influence on survival in the multivariate regression analysis were used to create a prognostic score. Binarization of the scoring parameters was based on the median, the discrimination values were then analyzed applying the Kaplan-Meier Method and Log-rank test. All tests were two-sided, statistical significance was assumed at a *p* < 0.05.

## Results

### Treatment and toxicities

We performed a total of 178 radioembolizations in 106 patients, including 12 whole-liver, 52 unilobar and 114 sequential lobar procedures. This resulted in a median number of 2 treatment sessions per patient (range: 1 – 5). Repeated radioembolizations of a specified liver volume with at least 3 sessions were limited to 8 patients. A median total activity of 1725 MBq (range: 200 – 3650 MBq) was administered per patient. The median tumor load was 15.7 %(range: 1.0 – 63.0 %).

No acute mortality was observed within 30 days post radioembolization. A total of 12 toxicities grade 3 or 4 according to CTCAE 4.02 were observed in 11 patients, see Table 2. Seven patients developed radiation induced liver disease (RILD) with ascites requiring paracentesis (*n* = 6), in one case associated with liver failure without tumor progression (*n* = 1). One patient displayed pleural effusion requiring thoracocentesis. Symptomatic gastric or duodenal ulcers occurred in three patients with subsequent endoscopic interventions. One patient underwent cholecystectomy after developing radiation induced cholecystitis.

**Table 2:** Treatment associated toxicities according to the Common Terminology Criteria for Adverse Events (CTCAE 4.03), a total of 12 major toxicities (grade 3 or 4) occurred in 11 patients

Major treatment related toxicities (grade 3 or 4), 12 events in 11 patients:		
Gastric or duodenal ulcer	*n* = 3	(3 %)
Pleural effusion	*n* = 1	(1 %)
Radiation induced cholecystitis	*n* = 1	(1 %)
Radiation induced liver disease (RILD) presenting with	*n* = 7	(7 %)
Refractory ascites	*n* = 6	(6 %)
Liver failure	*n* = 1	(1 %)

### Primary outcome and concomitant therapy

**Fig. 1:**
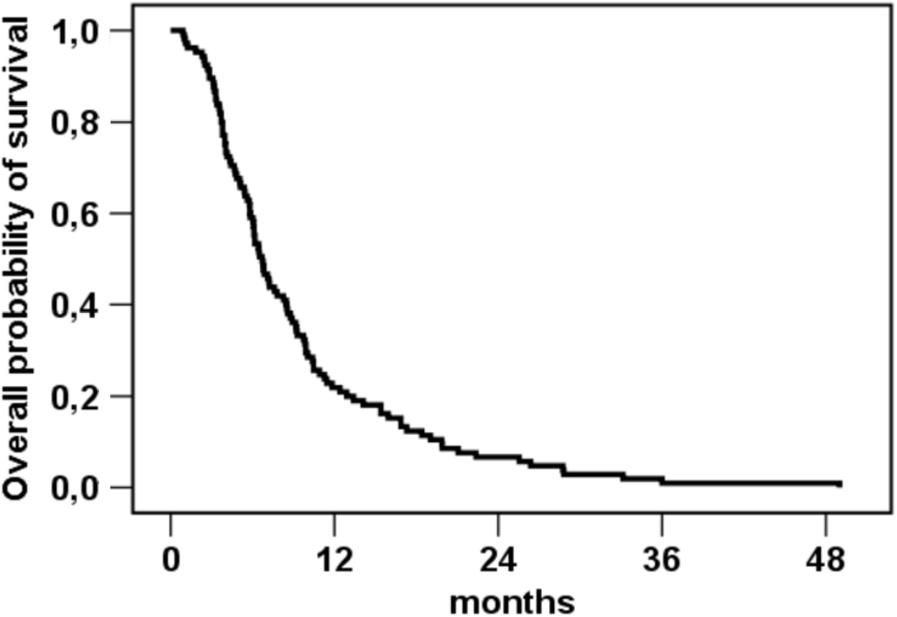
Kaplan-Meier estimation for overall survival after first radioembolization, all patients (*n* = 106). Since all patients had died by the time of analysis, no censored patients occur in the graph. Median overall survival (OS) was 6.7 months

Within the observation period, all patients deceased. Hence, no censored patients occur in our survival analyses. The median follow-up was 6.0 months (range: 1.0 – 48.0 months). The median overall survival of all 106 patients was 6.7 months after the first radioembolization as illustrated in the Kaplan-Meier survival curve (Fig. 1). The median progression free survival assessed by RECIST 1.1 was 3.5 months.In the follow-up period after Y90-radioembolization, 13 patients were given monotherapy with newly available antibodies (e.g. panitumumab). Another 9 patients received further cytotoxic chemotherapy following Y90 radioembolization.

### Regression analysis

At first, univariate stepwise Cox regression analysis was carried out for patient demographics and individual performance as well as tumor and treatment characteristics.

Regarding patient characteristics, a significant influence on patient survival was found for the Karnofsky index (median 80 %, range 60 – 100 %; *p* = 0.014), but not for age and sex. Prior resection or radiofrequency ablation, the type of systemic therapy and the number of chemotherapy lines before Y90 radioembolization had no significant influence on patient survival.

The hepatic tumor load (median 15.7 %, range 1.0 – 63.0 %) was found a significant factor (*p* < 0.001) while initial tumor staging, grading and extrahepatic manifestations had no significant influence. Furthermore, the serum level of specific tumor markers CEA (median 130.1 ng/ml, range 2.7 – 8713.3 ng/ml) and CA19-9 (median 192.7U/ml, range 0.3 - 32206.0 U/ml) were identified as prognostic factors (*p* = 0.002 and *p* < 0.001, respectively). Detailed parameters of radioembolization (e.g. whole-liver treatment, number of treatment sessions and administered activity of Y90) showed no significant influence. The results of the univariate analysis are summarized in Table 3.

**Table 3:** Cox regression of potential factors to predict survival

Cox regression	Hazard	(95 % CI)	Univariate P	Multivariate P
Age	1.02	(1.00–1.04)	0.239	
Sex‘	0.76	(0.50–1.14)	0.179	
Karnofsky index	0.98	(0.95–0.99)	0.014*	0.037**
Resection/RFA‘	0.90	(0.56–1.44)	0.658	
Oxaliplatin + 5-FU‘	1.44	(0.91–2.26)	0.118	
Irinotecan + 5-FU‘	1.41	(0.82–2.46)	0.216	
Capecitabine‘	1.24	(0.79–1.94)	0.344	
Bevacizumab‘	0.87	(0.58–1.30)	0.492	
Cetuximab‘	1.26	(0.85–1.87)	0.243	
Overall chemotherapy lines	1.14	(0.94–1.37)	0.179	
UICC staging	1.01	(0.79–1.30)	0.286	
Tumor grading	0.90	(0.64–1.28)	0.434	
Lung metastases‘	1.53	(0.85–2.74)	0.155	
Lymphatic metastases‘	1.60	(0.91–2.80)	0.100*	0.204
Bone metastases‘	2.51	(0.89–7.13)	0.083*	0.083
Hepatic tumor load	1.05	(1.03–1.06)	<0.001*	0.001**
CEA serum level	1.00	(1.00–1.00)	0.002*	0.023**
CA19-9 serum level	1.00	(1.00–1.00)	<0.001*	<0.001**
Bilobar Y90 RE‘	0.89	(0.45–1.77)	0.740	
Unilobar Y90 RE‘	0.74	(0.35–1.55)	0.426	
Sequential lobar Y90 RE‘	0.82	(0.40–1.66)	0.775	
Y90 RE sessions per patient	0.95	(0.72–1.24)	0.692	
Total activity per patient	1.00	(0.99–1.00)	0.276	

Results in the univariate analysis (*p < 0.1) were included in the multivariate analysis (**p < 0.05). Binary factors are marked (‘), other variables are ordinal or continuous

In a second step, all factors with a *p* ≤ 0.1 were included in a multivariate Cox regression. In this analysis, significant results were found for the Karnofsky index (*p* = 0.037), hepatic tumor load (*p* = 0.001) and serum levels of CEA and CA19-9 (*p* = 0.023 and *p* < 0.001, respectively). Concomitant bone or lymphatic metastases had no significant influence on the prognosis (*p* = 0.083 and *p* = 0.204, Table 3).

### Clinical score

CEA and CA19-9 serum levels, hepatic tumor load and Karnofsky index were further processed to form a prognostic score.

We binarized these prognostic factors approximating their median, identifying patients with:

- tumor load > 20 %,
- CEA level > 130 ng/ml and/or CA19-9 level > 200 U/ml,
- Karnofsky index < 80 %.

Each of these poor prognostics factors was attributed a single point. Complete data was available for 87 patients (82 %).

**Fig. 2:**
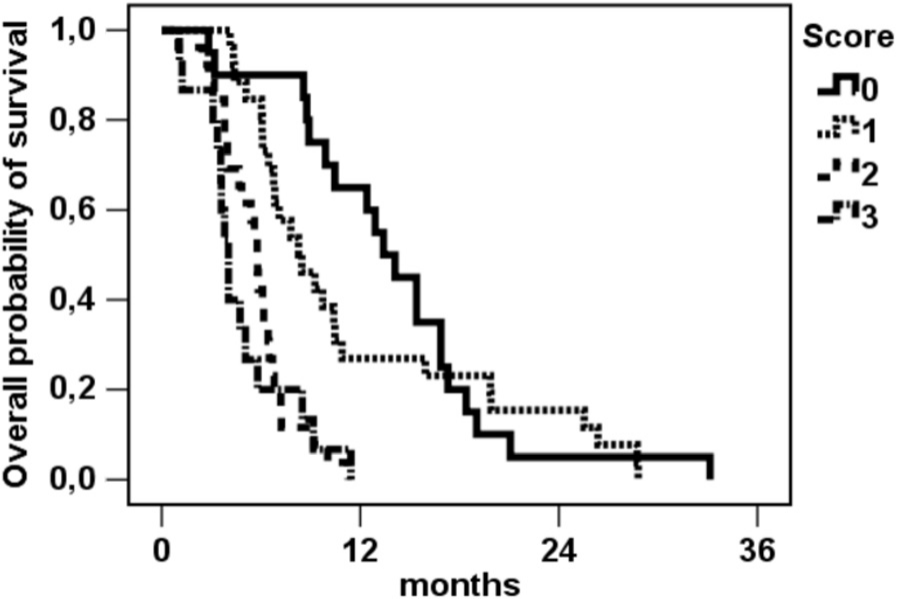
Kaplan-Meier estimation for overall survival after first radioembolization, strata by score points (*n* = 87). Since all patients had died by the time of analysis, no censored patients occur in the graph. Median OS was 13.4 months (0 points, *n* = 20), 8.3 months (1 point, *n* = 26), 5.8 months (2 points, *n* = 26) and 4.0 months (3 points, *n* = 15), *p* < 0.001

Corresponding median survival was 13.4 months for patients displaying 0 points (*n* = 20), 8.3 months with 1 point (*n* = 26), 5.8 months with 2 points (*n* = 26) and 4.0 months with 3 points (*n* = 15), respectively (see Fig. 2). The log-rank test confirmed a significant discrimination between the according patient groups (*p* < 0.001).

**Fig. 3:**
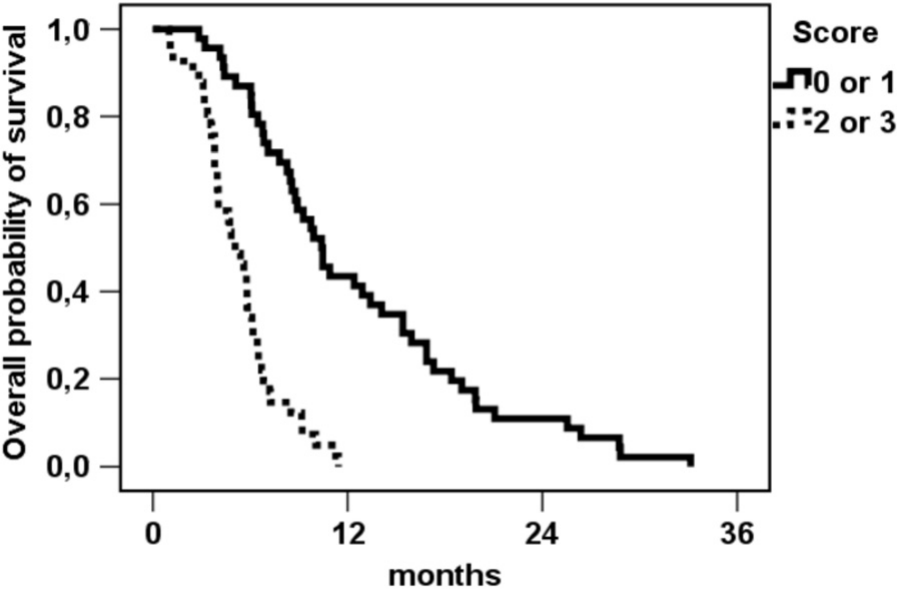
Kaplan-Meier estimation for overall survival (*n* = 87) after first radioembolization, groups of patients with survival benefit (score of 0/1) vs. no survival benefit (score of 2/3). Since all patients had died by the time of analysis, no censored patients occur in the graph. Median OS was 10.4 months (score 0 or 1) vs. 5.1 months (score 2 or 3), *p* < 0.001

When summarizing the groups of patients with 0 and 1 point versus 2 and 3 points, the according log-rank test demonstrated a survival of 10.4 months vs. 5.1 months (*p* < 0.001, see Fig. 3).

## Discussion

Y90 radioembolization has recently demonstrated its activity in treatment naïve colorectal liver only disease with a liver-only PFS improvement of 8 months when added to a FOLFOX first line treatment regimen [12]. However, the dominant proportion of patients admitted to Y90 radioembolization today presents in a salvage setting, with extensive liver tumor load, reduced performance status, chemorefractory disease, and a history of numerous and variable chemotherapy cycles [3, 4, 6]. Our own study contributes data to this patient selection with poor prognosis, with a median Karnofsky of 80 %, a tumor load of 20 %, and chemorefractory dise ase or patients refusing further chemotherapy as a results of toxicity. A total of 81 % patients presented with liver only disease, thirty-five patients (33 %) had undergone 2 lines and 62 patients (58 %) 3 or more lines of chemotherapy.

In this rather dismal patient cohort, the median overall survival of all patients undergoing Y90 radioembolization was 6.7 months. As such, the indication for Y90 radioembolization in our patient group may have been too aggressive, and the survival rate was worse than documented in other series of salvage mCRC Y90 radioembolization. Hendlisz et al. described a median survival of 10 months combining Y90 radioembolization and 5-FU (vs. 7.3 months in 5-FU only); Cosimelli et al. 12.6 months in a single arm cohort; Bester et al. 11.9 months versus 6.6 in control; and Seidensticker et al. 8.3 months versus 3.5 in control [3–6].

As systemic last line treatment, mitomycin C combined with capecitabine has been considered a well-tolerated salvage option for a long time, however associated with very low activity. Lim et al. published a cohort of 21 patients with a median survival of 6.8 months, commenting that there was no definitive contribution to increasing the patients overall survival [13]. Harba et al. reported 7.8 months overall survival in oxaliplatin and irinotecan refractory advanced mCRC [14]. More recently, the CORRECT study comparing cohorts receiving Regorafenib versus placebo reported outcomes of 6.4 versus 5.0 months (HR 0.77) in 760 randomized patients [15], however associated with ≥ grade 3 side effects hand-foot-skin-reaction, fatigue, diarrhea, hypertension and rash in 17 %,10 %, 7 %, 7 % and 6 %, respectively. For panitumumab monotherapy in KRAS wild type patients, van Cutsem et al. reported a reasonable antitumor activity in refractory patients with a median survival of 6.3 months [16].

Even though the pooled overall survival of all patients was poor in our own study, the scoring system derived out of this cohort holds promise for an improved patient selection. The score comprising of Tumor load, CA 19–9 and/or CEA, as well as Karnofsky (TuCK) discriminated two groups of patients with a median survival of 10.4 versus 5.1 months if each factor was attributed 1 point along with summing up patients with 0 and 1 versus 2 and 3 points. It is difficult to interpret whether the poor outcome of patients with 2 and 3 points reflects advanced disease stage or rather an aggressive tumor biology or poor performance status. The survival difference between the two groups (0 and 1 points vs. 2 and 3 points) truly mirrors a composite of multiple, independent factors representing stage, biology and individual patient performance, represented in our study by tumor load, clinical performance status and tumor markers. In addition, patients without these negative factors (TuCK 0) reached a median, overall survival of 13.7 months, which we consider highly favourable in a treatment refractory salvage situation. Interestingly, with the term “liver dominant disease” not clearly defined today, neither lung nor lymph node or bone metastases proved to have a significant impact on survival in our cohort.

Side effects grade 3 and 4 in the overall cohort of patients were limited to 11 of 106 patients (10 %, with 12 events total), indicating that Y90 radioembolization was of moderate toxicity in our patients. Hendlisz et al. in 2010 reported absence of any ≥ grade 3 event in 21 patients randomized to a combination therapy of 5-FU and Y90 radioembolization [5]. These data compare favourably to systemic salvage chemotherapeutic regimen such as by Regorafenib monotherapy with 232 of 500 patients experiencing ≥ grade 3 events, 85 of those discontinuing treatment for side effects (17 %) [15], and even to regimen considered well tolerable such as Capecitabine and mitomycin C with reports of 4 grade 3 or 4 toxicities in 19 patients [13], and 18 grade 3 events in 36 patients [16]. For panitumumab monotherapy, 2 % grade 4 toxicity have been reported, and the most frequent toxicity was skin toxicity [17].

In our patient cohort with a history of extensive chemotherapies and half of the patients displaying a hepatic tumor load >20 %, liver function after radioembolization, i.e. the subsequent development of radiation (radioembolization) induced liver disease (RILD or REILD), is of high interest according to the first description by Sangro et al.[18]. With 7 patients (6 %) displaying clinical symptoms which can be attributed to the development of RILD the incidence is lower than described previously with up to 20 % in a population of mixed tumor entities. We attribute this favorable outcome to the preventive effect of sequential lobar treatment at an interval of 4 to 6 weeks for the left and right liver lobe, as well as single lobar treatments if applicable [10]. In addition, RILD prevention by a drug regimen combining enoxaparin, ursodeoxycholic acid and pentoxiphylline for 8 weeks after treatment may have been beneficial [10, 19].

## Conclusion

A score based on Tumor load, CEA and/or CA19-9 serum level as well as the Karnofsky index demonstrated a close association with patient outcome after Y90 radioembolization in the salvage situation. Patients displaying more than 1 point may not benefit from liver directed Y90 radioembolization; in those patients alternative systemic treatments or best supportive care should be considered. In our population with severe liver dominant disease, lung, bone and lymph node metastases had no negative prognostic effect. The role of combined salvage Y90 radioembolization and systemic therapy remains unclear.

## Abbreviations

5-FU, 5-fluorouracil; CA19-9, Cancer antigen 19–9; CEA, carcino-embryonic antigen; CT, computed tomography; CTCAE, Common terminology criteria for adverse events; mCRC, metastatic colorectal cancer; MRI, magnetic resonance imaging; OS, overall survival; PFS, progression-free survival; RE, radioembolization; RECIST, response evaluation criteria in solid tumors; RILD, radiation induced liver disease; SPECT, single photon emission computed tomography; Tc-99 m MAA, Technecium-99 m macro-aggregated albumin; Y90, Yttrium-90
